# Three-dimensional image fusion guidance of percutaneous pulmonary valve implantation to reduce radiation exposure and contrast dose: A comparison with traditional two-dimensional and three-dimensional rotational angiographic guidance

**DOI:** 10.1007/s12471-016-0941-4

**Published:** 2016-12-13

**Authors:** S. Goreczny, T. Moszura, P. Dryzek, M. Lukaszewski, A. Krawczuk, J. Moll, G. J. Morgan

**Affiliations:** 1 Department of Cardiology, Polish Mother’s Memorial Hospital, Lodz, Poland; 2 Department of Radiology, Polish Mother’s Memorial Hospital, Lodz, Poland; 3 Medical University of Lodz, Lodz, Poland; 4 Department of Cardiology, Colorado Children’s Hospital & Department of Adult Congenital Cardiology, University of Colorado Hospital, Denver, CO USA

**Keywords:** PPVI, 3DRA, 3D guidance, VesselNavigator

## Abstract

**Introduction:**

Three-dimensional rotational angiography (3DRA) has been used in the guidance of various transcatheter therapies including percutaneous pulmonary valve implantation (PPVI). The most recently available 3D image fusion software (VesselNavigator, Philips) extends this technology to use pre-registered computed tomography or magnetic resonance imaging datasets, promising reductions in contrast and radiation exposure along with shorter procedural times.

**Methods:**

In this retrospective review, patients were assigned to three groups according to the mode of imaging guidance: two-dimensional angiography (2DA), 3DRA and VesselNavigator (VN) assisted valve implantation. Patient characteristics and catheterisation data were reviewed with a focus on contrast and radiation exposure, fluoroscopy, and procedural times.

**Results:**

Between July 2012 and June 2016, 21 patients underwent PPVI: 8 with 2D guidance, 6 patients with 3DRA and most recently 7 patients with VN assistance. Patents in the VN group received significantly less absolute and weight indexed contrast when compared with those with 2DA or 3DRA guided PPVI. Patients in the 2DA group received a significantly higher total dose area product radiation dose and air kerma in comparison with patients with 3DRA and VN guided intervention. Application of VN resulted in the shortest fluoroscopy time, although not statistically significant, and a significantly shorter study time when compared with 2DA.

**Conclusions:**

Utilisation of pre-intervention image manipulation with VesselNavigator for 3D guidance of PPVI results in a reduction in contrast and radiation exposure and study time as compared with traditional 2D guidance, and contrast usage as compared with 3DRA.

## Introduction

Over the last 15 years, percutaneous pulmonary valve implantation (PPVI) has become an effective and less invasive alternative to surgical right ventricle-to-pulmonary artery conduit replacement [1–3]. Promising early and mid-term outcomes have stimulated the search for new devices and techniques to offer this treatment to a wider patient population [4, 5]. Potential complications linked to this therapy, such as coronary artery compression, graft rupture or stent fracture, have required modifications of implantation protocols [6–8]. Despite non-invasive three-dimensional (3D) imaging being routinely performed in qualification and planning of PPVI, traditional two-dimensional (2D) imaging remains the mainstay for intervention guidance [1–8]. The development of fusion imaging software has led to the introduction of three-dimensional rotational angiography (3DRA) for the guidance of various transcatheter therapies, including PPVI [9–12]. The most recently available 3D i﻿m﻿a﻿g﻿e fusion software (VesselNavigator, Philips), which uses pre-registered computed tomography (CT) or magnetic resonance imaging (MRI) datasets, promises further reductions in contrast and radiation exposure along with shorter procedural times [13–15]. In this report we sought to describe our experience with three imaging modalities for guidance of PPVI.

## Methods

We performed a retrospective review of all percutaneous pulmonary valve implantations at the Polish Mother’s Memorial Hospital. Patients were assigned to three groups according to the mode of imaging guidance: two-dimensional angiography (2DA), 3DRA (Phillips Allura XPER FD20) and VesselNavigator (VN) assisted valve implantation. Patient characteristics and catheterisation data were reviewed with a focus on contrast and radiation exposure, fluoroscopy, and procedural times. For detailed comparison total contrast volume, dose area product, air kerma and fluoroscopy time were indexed to body weight.

### Procedural description

The majority of patients (19/21) had a contrast chest CT prior to discussion and qualification for PPVI on multidisciplinary meeting. The procedures were conducted un﻿der general anaesthesia, complying with the usual catheterisation techniques and standard PPVI protocol. 3DRA has been available at our institution since 2010, before commencement of the Melody valve implantation program. VesselNavigator was introduced in September 2015. Selection of imaging guidance was at the discretion of the operator.

### 3DRA

3DRA was performed with the following settings: a fully automated 4.1 s, 240˚ C-arm rotation from 120˚ right anterior oblique to 120˚ left anterior oblique with acquisition of 30 frames per second. To limit motion artefact the study was performed during an expiratory breath-hold. Initially we used full strength contrast and in the most recent patients we injected dilute contrast at a concentration of 60–70%. The delay between beginning of contrast injection and C‑arm rotation was set at 1 s. Manipulation to limit cardiac output during acquisition was not performed.

An unprocessed rotational dataset was manipulated on a dedicated workstation (Interventional Tools, Philips Healthcare) by the same operator for all patients. The reconstructed images were optimised by manual windowing and segmentation. The volume rendered 3D reconstruction was merged with live fluoroscopic images using the automated software for guidance of stent and valve positioning and implantation.

### VesselNavigator

**Fig. 1:**
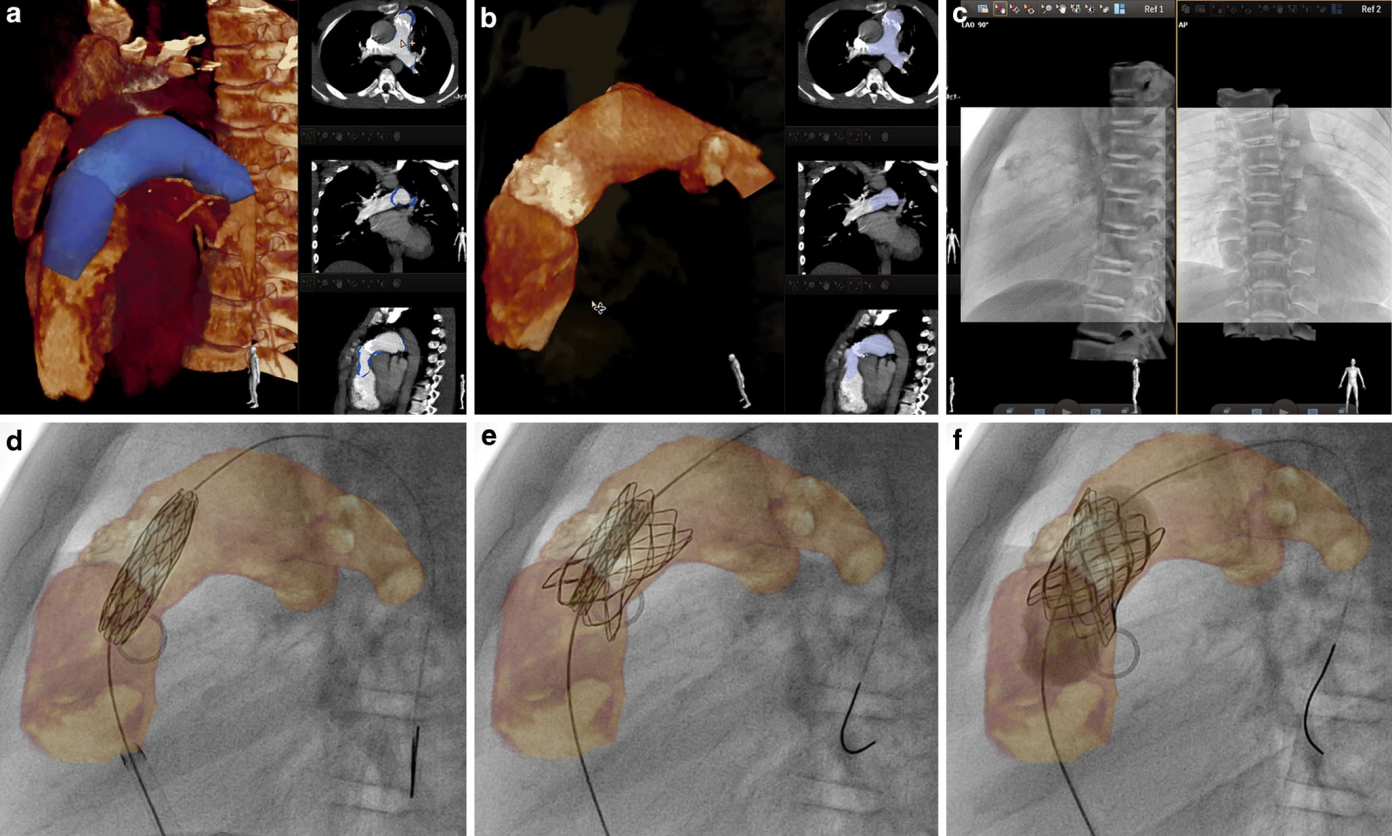
VesselNavigator assisted percutaneous pulmonary valve implantation. Automatic three-dimensional (3D) reconstruction (**a**, *left panel*) and row scans (*right panels*) from pre-registered computed tomography were manipulated to outline the conduit and the proximal pulmonary arteries (**b**). In addition to bony structures, heavy conduit calcifications were utilised to enhance manual 3D image fusion with live fluoroscopy (**c**). The 3D roadmap was utilised to guide creation of the landing zone (**d**) and subsequent implantation (**e**, **f**) of a Melody valve (Medtronic Inc.)

Application of VesselNavigator is based on four steps: segmentation, planning, registration and live guidance (Fig. 1; [13, 14]). In the first step, the application automatically creates a volume rendered 3D reconstruction from a raw CT or MRI dataset. Next, manual segmentation is performed by highlighting and selecting the desired region of interest on the 3D reconstruction or orthogonal MPRs. The second stage enables planning of the procedure by placing marking rings/points, taking measurements and selecting and storing the best angulations for the attempted intervention. The third stage includes fusion of live fluoroscopy and the manipulated 3D reconstruction. After storing fluoroscopy images in two, min 30° separated projections, vessel borders visualised with injection of a small amount of contrast medium or internal markers such as bony structures, shadow of the heart and great vessels, calcifications or previously implanted devices, serve as reference points for fusion. Finally live guidance of the procedure is performed with 3D roadmap overlaid and presented in several rendering modes with or without marking rings/points.

### Statistical analysis

Data analysis was performed using GraphPad InStat software (GraphPad Software, Inc., San Diego, CA, USA). Data are presented as frequency with percentage of the to﻿tal, median with range, or mean ± standard deviation, as appropriate. Student’s t‑test or a Mann-Whitney test, where indicated, was used for analysis. The level of statistical significance was set at *p* ≤ 0.05.

## Results

Between July 2012 and June 2016, 21 patients underwent PPVI: 8 with 2DA guidance, 6 patients with 3DRA and most recently 7 patients with VN assistance. Table 1 summarises patients clinical and procedural characteristics. Comparison of demographic data showed no difference between the three groups (Table 2). There were no significant differences in the number of previous interventions, pulmonary stenosis gradient, number of implanted stents, and repeat dilation after implantation.

**Table 1:** Clinical and procedural characteristics of all patients treated with PPVI

	Total (21)
Age (years)	13.8 (7.8–19.6)
Weight (kg)	46 (28–98)
BSA (m^2^)	1.5 (1.0–2.2)
*Diagnosis*	
Truncus arteriosus	8 (40%)
Tetralogy of Fallot	5 (25%)
Aortic stenosis	5 (25%)
Other	2 (10%)
Number of previous RVOT intervention	9 (45%)
Pulmonary stenosis gradient (mm Hg)	32 (6–100)
Number of stents placed	27 (1–3)
Post-dilation	7 (35%)
*Ensemble size*	
18 mm	2 (10%)
20 mm	10 (50%)
22 mm	8 (40%)

*PPVI* percutaneous pulmonary valve implantation, *BSA* body surface area, *RVOT* right ventricle outflow tract

**Table 2:** Comparison of selected demographic and clinical data of PPVI guided with 2DA, 3DRA and VN

	Total (*n* = 21)	2DA (*n* = 8)	3DRA (*n* = 6)	VN (7)	*P*
Age (years)	13.8 (7.8–19.6)	14 (9.7–19.6)	13.8 (12.3–17.6)	11.2 (7.8–16.1)	2D vs 3DRA, *p* = 0.89 2D vs VN, *p* = 0.29 3DRA vs VN, *p* = 0.19
Weight (kg)	46 (28–98)	53.5 (28–98)	49.3 (42.8–52.0)	40 (29–56)	2D vs 3DRA, *p* = 0.65 2D vs VN, *p* = 0.20 3DRA vs VN, *p* = 0.07
BSA (m^2^)	1.5 (1.0–2.2)	1.7 (1.0–2.2)	1.5 (1.4–1.6)	1.2 (1.1–1.6)	2D vs 3DRA, *p* = 0.59 2D vs VN, *p* = 0.07 3DRA vs VN, *p* = 0.06
PS gradient (mm Hg)	32 (6–100)	34.5 (12–51)	25 (6–75)	41 (20–100)	2D vs 3DRA, *p* = 0.99 2D vs VN, *p* = 0.28 3DRA vs VN, *p* = 0.40
Number of stents placed	30 (1–3)	11 (1–2)	10 (1–3)	9 (1–2)	2D vs 3DRA, *p* = 0.43 2D vs VN, *p* = 0.73 3DRA vs VN, *p* = 0.32
Post-dilation	7 (35%)	3 (37.5%)	1 (17%)	3 (43%)	2D vs 3DRA, *p* = 0.79 2D vs VN, *p* = 0.83 3DRA vs VN, *p* = 0.68

*PPVI* percutaneous pulmonary valve implantation, *2DA* two-dimensional angiography, *3DRA* three-dimensional rotational angiography, *VN* VesselNavigator, *BSA* body surface area, *PS* pulmonary stenosis

**Fig. 2:**
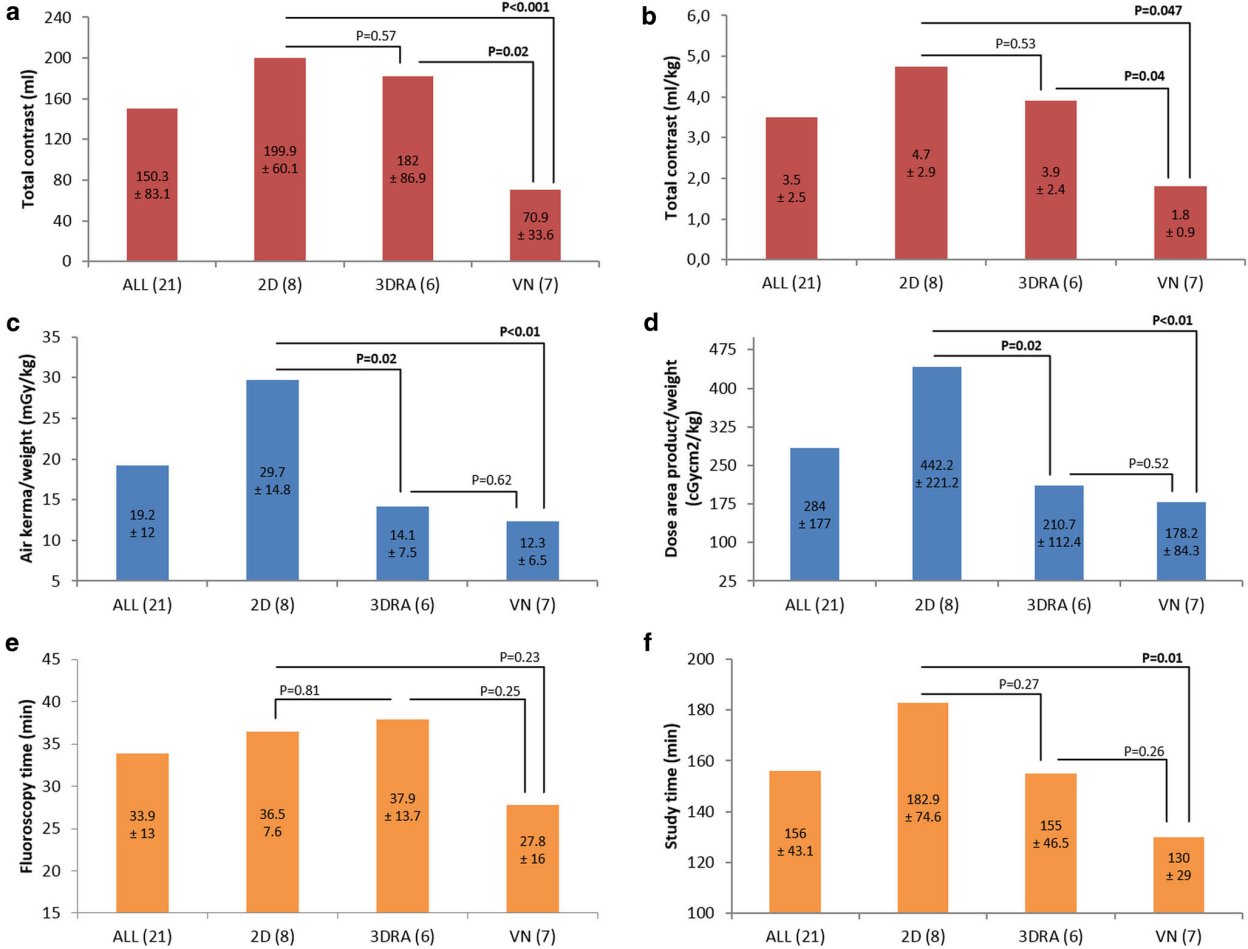
Comparison of absolute (**a**) and weight adjusted (**b**) contrast usage, weight adjusted air kerma (**c**) and dose area product (**d**), fluoroscopy (**e**) and study times (**f**) between two-dimensional angiography (2DA), three-dimensional rotational angiography (3DRA) and VesselNavigator (VN) guided percutaneous pulmonary valve implantation

VN patients received the lowest contrast dose (1.5 ml/kg, 1.1–3.6), significantly less than those with 3DRA (4.7 ml/kg, 1.5–6.3; *p* < 0.05) or 2DA guidance (3.8 ml/kg, 1.0–10.7; *p* < 0.05) (Table 3; Fig. 2). There was no significant difference in absolute or weight adjusted contrast usage between 2D and 3DRA guided PPVI.

**Table 3:** Comparison of contrast usage, radiation exposure, fluoroscopy and study times between PPVI performed with 2DA, 3DRA and VN guidance

		Total (*n* = 21)	2DA (*n* = 8)	3DRA (*n* = 6)	VN (*n* = 7)	*P*
Total contrast	ml	140 (40–300)	200 (99–300)	200 (78–300)	56 (40–130)	2D vs 3DRA, *p* = 0.57 **2D vs VN, ** ***p*** **< 0.001** **3DRA vs VN, ** ***p*** **= 0.02**
	ml/kg	3.5 (1–10.7)	3.8 (1.0–10.7)	4.7 (1.5–6.3)	1.5 (1.1–3.6)	2D vs 3DRA, *p* = 0.53 **2D vs VN, ** ***p*** **= 0.047** **3DRA vs VN, ** ***p*** **= 0.04**
Air kerma	mGy	736.9 (122–1665)	1191 (900.1–1665)	727 (400.1–1024.6)	450 (122–1293)	**2D vs 3DRA, ** ***p*** **< 0.01** **2D vs VN, ** ***p*** **< 0.01** 3DRA vs VN, *p* = 0.49
	mGy /kg	17 (3.9–42.5)	31.9 (16.4–42.5)	14 (8.9–21.6)	10.1 (3.9–23.1)	**2D vs 3DRA, ** ***p*** **= 0.02** **2D vs VN, ** ***p*** **< 0.01** 3DRA vs VN, *p* = 0.62
Dose area product	cGycm^2^	10980.3 (1507–24808.5)	17745.9 (13411.2–24808.5)	10832.3 (5961.2–15265.9)	6498.1 (1507–16694.1)	**2D vs 3DRA, ** ***p*** **< 0.01** **2D vs VN, ** ***p*** **< 0.001** 3DRA vs VN, *p* = 0.34
	cGycm^2^/kg	253.1 (48.6–632.6)	475.9 (243.8–632.6)	208.3 (132.5–321.4)	159.1 (48.6–298.1)	**2D vs 3DRA, ** ***p*** **= 0.02** **2D vs VN, ** ***p*** **< 0.01** 3DRA vs VN, *p* = 0.52
Fluoroscopy time	min	33.6 (9.3–55.3)	34.8 (29.5–51.5)	37.4 (21.5–55.3)	23.2 (9.3–53.5)	2D vs 3DRA, *p* = 0.81 2D vs VN, *p* = 0.23 3DRA vs VN, *p* = 0.25
	min x kg	1522.1 (288.3–3235)	1620.9 (1149.1–3235)	1689.8 (965.7–2873)	860 (288.3–2996)	2D vs 3DRA, *p* = 0.86 2D vs VN, *p* = 0.21 3DRA vs VN, *p* = 0.19
Study time (min)		145 (90–240)	190 (135–240)	137.5 (110–215)	125 (90–175)	2D vs 3DRA, *p* = 0.27 **2D vs VN, ** ***p*** **= 0.01** 3DRA vs VN, *p* = 0.26

*PPVI* percutaneous pulmonary valve implantation, *2DA* two-dimensional angiography, *3DRA* three-dimensional rotational angiography, *VN* VesselNavigator

Two-dimensional guidance resulted in significantly higher dose area product (DAP) (17,745.9 cGycm^2,^13,411.2–24,808.5) compared with 3DRA (10,832.3 cGycm^2^, 5961.2–15,265.9; *p* < 0.05) or VN (6498.1 cGycm^2^, 15,07.0–16,694.1; *p* < 0.05) assisted intervention. When adjusted to body weight, the 2DA group received the highest DAP (475.9 cGycm^2^/kg, 243.8–632.6) compared with 3DRA (208.3 cGycm^2^/kg, 132.5–321.4; *p* < 0.05) or VN (159.1 cGycm^2^/kg, 48.6–298.1; *p* < 0.05). The VN group received lower absolute and weight adjusted DAP compared with the 3DRA group; however, the differences were not statistically significant.

Patients in the 2DA group received significantly higher total and air kerma (1191 mGy, 900.1–1665), in comparison to those with 3DRA (727 mGy, 400.1–1024.6; *p* < 0.05) or VN (450 mGy, 122–1293; *p* < 0.05). When adjusted to patient weight, the 2DA group (31.9 mGy/kg, 16.4–42.5) was exposed to significantly higher air kerma when compared with 3DRA (14 mGy/kg, 8.9–21.6; *p* < 0.05) or VN group (10.1 mGy/kg, 3.9–23.1; *p* < 0.05).

Application of VN resulted in the shortest fluoroscopy time (23.2 min, 9.3–53.5) when compared with 3DRA (37.4 min, 21.5–55.3) or 2DA (34.8 min, 29.5–51.5); however, the differences were not statistically significant. The same observation was true for weight-fluoroscopy time.

Patients in the VN group had significantly shorter procedural times (125 min, 90–175) when compared with those in the 2DA group (190 min, 135–240; *p* < 0.05). VN procedural times also tended to be shorter than those in the 3DRA group (137 min, 110–215), but this difference was not statistically significant.

## Discussion

Three-dimensional imaging has become increasingly popular in the planning and guidance of cardiac catheterisations. Until recently, 3DRA has been the most frequently used tool to generate a volumetric dataset and reconstruct it to a 3D roadmap that could be used for navigation [9–12]. It has been shown to provide superior vascular visualisation, including complex spatial relationships unavailable with 2DA, and facilitate interventions in various congenital and structural lesions. However, limited data are available on the use of 3DRA in PPVI and even less is reported on pre-registered 3D images in this context. As non-invasive imaging of the right ventricular outflow tract (RVOT), pulmonary arteries and coronary arteries is part of the routine protocol for PPVI, it seems natural to re-produce the previous dataset for 3D mapping during the intervention. Advances in image fusion software allow direct 2D-3D registration of live fluoroscopy with rendered 3D imaging from CT or MRI scans, promising a reduction in contrast and radiation exposure along with shorter procedural times [14, 15].

In this study, for the first time, we compared three imaging modalities for guidance of PPVI. When compared with traditional 2DA, VN assistance resulted in significantly lower contrast utilisation, radiation exposure and total study time with a tendency towards shorter fluoroscopy time. Our data reinforce results described by Tacher et al. for endovascular aneurysm repair guided with 2DA, 3DRA and fusion software similar to VN but based on 3D-3D registration [16]. More recently Stangenberg et al. reported the first application of VN for aortic aneurysm repair. They compared VN and 2DA guided standard endovascular repair, achieving superior radiation and contrast dose parameters with VN [13]. We were not able to show a statistically significant reduction in fluoroscopy time, despite recording the shortest minimum and median fluoroscopy times in the VN group. This may have been due to an uneven distribution of technically challenging cases, which may have required prolonged fluoroscopy regardless of the type of imaging.

Nguyen at al. analysed 81 PPVIs including 29 with 3DRA guidance [12]. They were able to show that 3DRA does not increase radiation exposure, despite patients in the 3DRA group having more additional interventions. Although based on a smaller patient population, our experience concurs that 3DRA provided lower DAP and air kerma compared with 2DA.

Haddad at al. performed comparison of radiation and contrast exposure for 3DRA with customised low dose radiation protocol and 2DA [11]. In the group of 100 control matched interventions, including 18 PPVIs, they reported similar total radiation and contrast doses. This reflects our observation that although utilisation of a 3D roadmap limits or even negates contrast injections during stent/valve positioning, it requires a relatively large contrast load to obtain the initial dataset.

In the context of contrast utilisation, VN assistance resulted in the lowest volumes used in the entire group. In fact, already in our early experience, reliable 3D roadmaps allowed us to avoid RVOT contrast injections prior to stent implantation in the majority of VN guided valve implants.

Lower contrast usage was the only significant difference between 3DRA guidance and VN guidance in our study. Although not statistically significant, the VN group had lower radiation exposure, fluoroscopy and procedural times than 3DRA. We need to acknowledge, however, that in several VN assisted interventions 3DRA was performed at the end of the study to visualise the final outcome. This might confound results of the comparison but limited population size hindered powerful statistical analysis of the subgroups. Both 3D techniques resulted in significant lower radiation exposure as compared with traditional 2DA.

This manuscript represents early clinical experience with a complex new technique. Our hope and expectation is that as we move along the learning curve we will see a more demonstrable difference in favour of VN fusion imaging with respect to all the parameters examined in our study.

## Limitations

This is a single-centre, non-randomised retrospective study. Due to the limited patient population we did not analyse subgroups of patients with previous pre-stenting, those with multiple 3DRAs or with a combination of imaging techniques. Although the 3D roadmaps were prepared by the same operator, valve implantations were performed by three operators with different experience. Air kerma and DAP were used as a predictors of deterministic and stochastic effects, respectively; however, we did not have the capability to convert DAP into effective dose.

## Conclusions

Integration of pre-intervention imaging using the VesselNavigator for 3D guidance of PPVI results in a significant reduction in contrast, radiation exposure and study time, compared with traditional 2DA. VN assistance also led to significantly lower contrast usage. The radiation exposure tended to be lower than with 3DRA, but this difference did not reach statistical significance. Our initial experience provides a proof of concept. We require a large multi-centre collaborative study to fully evaluate this technology.

## References

[1] Khambadkone S, Coats L, Taylor A (2005). Percutaneous pulmonary valve implantation in humans: results in 59 consecutive patients. Circulation.

[2] Eicken A, Ewert P, Hager A (2011). Percutaneous pulmonary valve implantation: two-centre experience with more than 100 patients. Eur Heart J.

[3] Biernacka EK, Rużyłło W, Demkow M (2015). Transcatheter pulmonary valve implantation in patients with right ventricular outflow tract dysfunction: early and mid-term results. J Invasive Cardiol.

[4] Promphan W, Prachasilchai P, Siripornpitak S, Qureshi SA, Layangool T (2016). Percutaneous pulmonary valve implantation with the Venus P‑valve: clinical experience and early results. Cardiol Young.

[5] Demkow M, Rużyłło W, Biernacka EK (2014). Percutaneous Edwards SAPIEN(™) valve implantation for significant pulmonary regurgitation after previous surgical repair with a right ventricular outflow patch. Catheter Cardiovasc Interv.

[6] Fraisse A, Assaidi A, Mauri L (2014). Coronary artery compression during intention to treat right ventricle outflow with percutaneous pulmonary valve implantation: incidence, diagnosis, and outcome. Catheter Cardiovasc Interv.

[7] Goreczny S, Eicken A, Ewert P, Morgan GJ, Fratz S (2014). A new strategy to identify potentially dangerous coronary arterial patterns before percutaneous pulmonary valve implantation. Postepy Kardiol Interwencyjnej.

[8] Cardoso R, Ansari M, Garcia D, Sandhu S, Brinster D, Piazza N (2016). Prestenting for prevention of melody valve stent fractures: a systematic review and meta-analysis. Catheter Cardiovasc Interv.

[9] Goreczny S, Dryzek P, Moszura T (2012). Rotational angiography in monitoring of covered CP stent implantation in patient with critical aortic coarctation and patent ductus arteriosus. Kardiol Pol.

[10] Goreczny S, Morgan GJ, Dryzek P (2016). Live 3D image overlay for arterial duct closure with Amplatzer Duct Occluder II additional size. Cardiol Young.

[11] Haddad L, Waller BR, Johnson J (2016). Radiation protocol for three-dimensional rotational angiography to limit procedural radiation exposure in the pediatric cardiac catheterization lab. Congenit Heart Dis.

[12] Nguyen HH, Balzer DT, Murphy JJ, Nicolas R, Shahanavaz S (2016). Radiation exposure by three-dimensional rotational angiography (3DRA) during trans-catheter melody pulmonary valve procedures (TMPV) in a pediatric cardiac catheterization laboratory. Pediatr Cardiol.

[13] Stangenberg L, Shuja F, Carelsen B, Elenbaas T, Wyers MC, Schermerhorn ML (2015). A novel tool for three-dimensional roadmapping reduces radiation exposure and contrast agent dose in complex endovascular interventions. J Vasc Surg.

[14] Goreczny S, Dryzek P, Moszura T (2016). Novel 3‑dimensional image fusion software for live guidance of percutaneous pulmonary valve implantation. Circ Cardiovasc Interv.

[15] Goreczny S, Dryzek P, Moszura T (2016). Use of pre-intervention imaging with a novel image fusion software for guidance of cardiac catheterisation in a patient with pulmonary atresia and major aortopulmonary collaterals. Cardiol Young.

[16] Tacher V, Lin M, Desgranges P (2013). Image guidance for endovascular repair of complex aortic aneurysms: comparison of two-dimensional and three-dimensional angiography and image fusion. J Vasc Interv Radiol.

